# Clinical Memoranda on Cough

**Published:** 1903-07

**Authors:** Leopold F. W. Haas, G. A. De Santos Saxe

**Affiliations:** Attending Physician to the Children’s Department of the German Poliklinik; Visiting Physician to the Messiah Home for Children, New York; Formerly Attending Physician to the Children’s Department of the German Poliklinik, New York


### Clinical Memoranda on Cough.

By LEOPOLD F. W. HAAS, M. D.,
Attending Physician to the Children's Department of the German Poliklinik; Visiting Physician to the Messiah Home for Children, New York.

AND

G. A. de SANTOS SAXE, M. D.,
Formerly Attending Physician to the Children's Department of the German Poliklinik, New York.

In 1898 Dreser[1], while looking for a morphine derivative that would prove superior to codeine as regards its influence upon the respiration, found that the diacetic acid ester of morphine, heroin [$C_{17}H_{17}:(O.OC.CH_3)_2:NO$], was a substance which could be described as an ideal respiratory sedative.

Since the publication of Dreser's article, his observations have been confirmed and amplified by trustworthy observers in all parts of the world, and the introduction of heroin gave medicine a drug that was destined to remain as a remedy of acknowledged worth, of undoubted trustworthiness, and of marked advantages over its predecessors, the sedative narcotics.

The physiological action of heroin has now been thoroughly studied, both by experiments upon animals and by clinical observations. Dreser, whose comparative studies of the action of the various morphine derivatives as respiratory sedatives have become classical, was the first to establish the fact that heroin had a more profound sedative action on the respiration than morphine, and that it was ten times more powerful in this respect, dose for dose, than codeine, while the fatal dose of heroin was ten times larger than that of the last-named alkaloid. These quantitative relations of heroin to the other morphine derivatives placed it in a class of its own. The principal effect of heroin is to reduce the frequency of the respiration, but this takes place, unlike the case of other respiratory sedatives, without any loss of oxygenation to the lung alveoli, for the less frequent respirations after a dose of heroin are deeper and fuller, the force of the inhalations being increased. Therefore, while the intervals between the inspirations are lengthened, and the duration of the inspiration is increased, the force of the act of breathing is so enhanced that there is a more perfect aeration of the pulmonary alveoli than before taking the drug. This property of heroin of increasing the aeration of the lungs may at once be recognised as of the utmost importance in the treatment of catarrhal conditions of the respiratory tract, for it is essential in these diseases that the secretions be not allowed to stagnate in the lungs and bronchi, but that a free access of air should take place to the lung surface so that the healing of inflammatory lesions may be promoted.

The general physiological effects of heroin have also been studied, and it was found that the administration of moderate doses of this drug produces a reduction in the consumption of oxygen by the body, and correspondingly diminishes the amount of carbon dioxide eliminated. This property is valuable in two sets of conditions—namely, first, in states accompanied by dyspnea, or air hunger; and, second, in febrile conditions of a hectic type, where heroin diminishing oxidation, lessens heat production and lowers the temperature. The reduction of the respiratory activity which is produced by heroin, is therefore compensated by a fuller aeration of the lungs, and by a lessened demand for oxygen. Besides, it must be noted, that heroin does not in any appreciable degree diminish the sensitiveness of the respiratory center to the chemical balance of oxygen and carbon dioxide in the blood, and does not, like codeine, increase the reflex irritability of the respiratory center in the presence of an obstruction in the air passages.

The action of heroin on the circulation is perfectly satisfactory, from the viewpoint of its desirability as a respiratory sedative. Only excessive doses affect the pulse rate and the blood pressure, and after fatal doses the heart still continues to beat after respiration has for some time ceased to be appreciable. Death, therefore, takes place by respiratory paralysis in these cases. Moderate, and even large, doses of heroin do not produce any disturbance on the part of the circulation.

The unpleasant symptoms following the use of morphine, codeine, and other narcotics, are totally lacking in the use of heroin. No headache, nausea, vomiting and depression, occurs after its use. The drug is practically devoid of hypnotic effect, and its prolonged use does not create a habit, nor does it produce constipation. In all these respects heroin is superior to morphine and codeine. Some authors have, however, reported cases in which moderate doses of heroin have been followed by a feeling of lassitude[5] and of dizziness, but we have never seen such cases among a large number in which we have used heroin. In our experience with heroin, which began in the winter of 1898-99, we have found a satisfactory respiratory sedative, and an efficient remedy in the treatment of coughs, both in children and adults.

The best mode of administering heroin so as to bring its good qualities into full play has occupied us for some time, and we have tried successively the various methods of administration recommended by writers on this subject, such as Floret[1], Manges[2], Freudenthal[5], North[7], Einhorn[11], Wetherby[17], Medea[19], Hyams[32], Martinson[38], and others.

We found, however, that the most satisfactory way of administering heroin was in a glycerine solution, combined with expectorants and stomachics. Glycerine seems to serve as the best medium for blending the properties of heroin with those of the other drugs used in the combination. The expectorants which we added, almost from the first, to heroin, seemed to enhance the rapidity of its action, causing secretion to be coughed out in a painless, easy manner, thus ridding the respiratory mucosa of irritating foreign matter and adding to the respiratory capacity of the lungs. The idea of adding stomachics and bitters was to counteract any effects that heroin, or the other drugs in the formula, may have upon sensitive stomachs when the remedy is administered for a length of time.

A number of combinations were tried in this way, until two years ago, when we began to use glycoheroin (Smith), a preparation which is composed of a glycerine solution of heroin with the addition of expectorants and stomachics,—just on the lines on which our former prescriptions were based. The success which we had had in treating cough with heroin in such combinations gave us hope that glycoheroin would give good results, and we decided to test it thoroughly, as regards its value as a mode of administering heroin, giving it to the exclusion of other remedies, wherever indicated in a series of cases. The results of our experience with glycoheroin are presented below.

Glycoheroin is described as a dark amber-colored liquid, which is perfectly clear, of a density approaching that of pure glycerine, of an agreeable aromatic odor, and a sweet taste with a slight suggestion of bitterness. Its formula is as follows:

| | | | |
|---|---|---|---|
| ℞ | Heroin hydrochloride ......... | gr. $\frac{1}{16}$ | 0.00375 gm. |
| | Ammonium hypophosphite .... | gr. iii. | 0.18 gm. |
| | White pine bark ............. | gr. 3½ | 0.21 gm. |
| | Balsam of tolu............... | gr. ¼ | 0.015 gm. |
| | Hyoscyamus.................. | gr. 1 | 0.06 gm. |
| | Glycerine, enough to make .... | ʒj | 4.0 gm. |

Each fluid drachm of glycoheroin therefore contains exactly 1-16th of a grain of heroin hydrochlorate. The dose chosen represents an average amount given to adults, every two, three or four hours. In children we have made a practice of diluting glycoheroin with various vehicles, usually either with glycerine or with simple syrup; or, when occasion required, we have taken the opportunity offered by the dilution to add other drugs, as some of the prescriptions given below will indicate. In children the dose ranges from one to thirty drops. The following table, from Lowenthal[34], may be found useful in this connection:

| | | | | |
|---|---|---|---|---|
| Aged | 1 month............ | grain 1-300 | maximum | dose. |
| " | 2 months........... | " 1-275 | " | " |
| " | 3 " ........... | " 1-250 | " | " |
| " | 4 " ........... | " 1-240 | " | " |
| " | 6 " ........... | " 1-220 | " | " |

| | | | |
|---|---|---|---|
| Aged 8 months | grain | 1-200 | maximum dose. |
| " 10 " | " | 1-150 | " " |
| " 12 " | " | 1-125 | " " |
| " 2 years | " | 1-50 | " " |
| " 3 " | " | 1-36 | " " |
| " 4 " | " | 1-30 | " " |
| " 5 " | " | 1-25 | " " |
| " 6 " | " | 1-20 | " " |
| " 7 " | " | 1-18 | " " |

Ten drops of glycoheroin represent 1-96th of a grain of heroin hydrochloride, so it is easy to calculate the dosage for children and to dilute the dose to a teaspoonful. A few words must be said as regards ammonium hypophosphite, which figures in the above formula. It is a salt that is comparatively rarely used, but one that is superior as an expectorant to the other ammonium salts in the mildness of its action and the absence of irritation, either to the larynx, the bronchi or the stomach, following its use. The virtues of ammonium hypophosphite have not been generally recognised to the extent which they deserve.

The other constituents of glycoheroin are remedies of such thoroughly accepted standing, that they need no comment, for the astringent properties of white pine and the soothing effect of balsam of tolu are known to everybody. Hyoscyamus is added as a stomachic. Glycerine is undoubtedly the best vehicle that can be selected for these drugs; it enables the combination to remain perfectly unchanged for a long time, and lacks the fermentative tendencies of sugar and syrup.

We have observed a series of 20 cases, which are the last 20 treated with heroin that have come under our observation, in 13 of which we wish to record in detail our results just as they were noted, without attempting to exaggerate on the one hand, nor to underestimate on the other, the effects produced by glycoheroin on the leading symptom—cough.

The histories have been condensed as far as possible, but in each enough has been given to show the nature of the case, the sequence of events, and the quality of the results obtained.

REPORT OF CASES.

Case I.—*Tuberculosis.*—E. O., aged 40 years; was suffering from cough for several years. He had severe pains in the back and side; a troublesome cough, with mucopurulent expectoration, and was hoarse at times. The physical examination showed coarse as well as fine râles and friction sounds. Tubercle bacilli were found in the sputum. The diagnosis was pulmonary tuberculosis.

On October 4th, glycoheroin was prescribed in drachm doses, every two hours. On the 8th, the patient felt much better; his cough was loose and less frequent, and his pains were not so severe. The medication was continued every three hours. On October 11th, the improvement continued. He coughed very little, only in the morning, was not so hoarse and had hardly any pains. Glycoheroin was continued three times daily. On the 13th he went to work, in spite of my warning to the contrary, and had a severe hemoptysis on the following day. Rest was ordered, and glycoheroin was continued every four hours, no other drugs being given. On the 22d he felt well, still had cough in the morning, with expectoration, but there was hardly any cough during the day. He slept well and had a good appetite.

November 8th. He has been feeling well and has not been troubled with cough. There were no bacilli found in the sputum. Throughout the time of observation his temperature had varied from 99° to 102° F. in the afternoon. On November 8th the temperature was normal, and had been for a week.

Case II.—*Tuberculosis.*—S. A., aged 38 years; has been ill since March, 1901. He had been coughing and had had a mucopurulent expectoration and severe pains in the back and sides since that time. His appetite had been poor, his sleep disturbed and restless owing to the cough, and he has had pains in the stomach, especially after meals, probably due to the enormous amount of cod liver oil and creosote which he had been taking. He had rapidly lost flesh and strength and had been operated upon by one of us (L.F.H.) for a tuberculous fistula in ano with a resulting permanent healing. Numerous tubercle bacilli were found in his sputum. The diagnosis was general tuberculosis.

On October 6th, having been disappointed in the effects of creosote, guaiacol, and the like, upon his cough, and knowing his to be a hopeless case, we decided to give him glycoheroin in drachm doses every two hours. On October 14th, one week later, the patient felt better, his breathing was easier, his cough looser and not so frequent. His appetite had improved and there were no digestive disturbances. The medication was continued every three hours. On October 19th, the patient ceased to take glycoheroin, thinking himself sufficiently improved to go without medicine. On the 21st he presented himself again, feeling considerably worse, the cough again having become more severe and the pains having increased. The same medication was ordered, every three hours. On the 25th the patient once more felt well, the cough troubled him only in the morning and the expectoration was mucopurulent, but he had not slept so well.

On November 6th he felt better and could sleep fairly well, but he still coughed in the morning. He admitted, however, that he felt quite "easy."

NOTE—This is a case in which we could only hope to moderate the symptoms, which the glycoheroin did. The disease is slowly but surely progressing.

Case III.—*Subacute Bronchitis (Tuberculosis?).*—C. S., aged 30 years; had been coughing for about two weeks; felt weak and did not sleep well. He had expectorated some clear, red blood, but there were no signs of tuberculosis found in his chest and no bacilli in his sputum, which was scant and mucoid in character.

On October 6th, glycoheroin was given in drachm doses every three hours. In four days his cough had stopped entirely; he felt easier and slept well. His breathing had also become easier. He was sent to the mountains for two weeks and returned in good health, having gained seven pounds.

The diagnosis in this case was probably a simple bronchitis of the subacute type.

Case IV.—*Chronic Bronchitis and Asthma.*—Mrs. R. V., aged 42 years; has had cough with expectoration, accompanied by dyspnea, every winter for the past eight or nine years. No tubercle bacilli were found in her sputum.

On October 20th, a drachm of glycoheroin was administered every three hours. On the 25th the patient was greatly improved. Her cough had diminished considerably. It was more marked during the day and scarcely present during the night. Her dyspnea was greatly relieved. The medication was continued, glycoheroin being given every four hours. On November 3d, the improvement had steadily continued, and her cough was much easier and less frequent.

NOTE—In this case the physiological action of heroin was well illustrated, the patient's breathing showing a diminution in the rate and an increase in the depth of the respirations.

Case V.—*Chronic Bronchitis and Asthma.*—P.H., 55 years old; had been suffering with acute attacks of asthma and bronchitis every winter for the past few years, and has been coughing more or less constantly during this time.

On October 19th, glycoheroin was given in drachm doses every three hours. On the 23d his cough had become looser, he slept sounder and more quietly but he still coughed considerably. On the 26th no improvement was noted; in fact his cough had become worse and his breathing harder. Glycoheroin was discontinued. The result was unsatisfactory in this case.

Case VI.—*Laryngitis and Pharyngitis.*—Miss B. P., aged 19 years; complained of a short, hacking cough, and was slightly hoarse. Her pharynx and larynx were congested and she had adenoid vegetations. Her tonsils had been removed some time ago. There were no signs of disease in her lungs. A drachm of glycoheroin was given every two hours. The patient complained of feeling drowsy, but coughed continually. The result in this case was unsatisfactory, probably because the patient did not take the medicine regularly.

Case VII.—*Bronchitis and Laryngitis.*—L. L., a boy 3 years old; had a croupy cough, which was worse at night. Glycoheroin was given in five-drop doses, every three hours in the daytime and every two hours towards evening and at night. The hoarseness disappeared entirely in three days, while the cough lasted a few days longer.

Case VIII.—*Acute Bronchitis.*—Miss F. H., 15 years old; was suffering from a dry and irritating cough for three days. A half drachm of glycoheroin every three hours was given and the cough disappeared entirely in four days.

Case IX.—*Tuberculosis.*—P. E., a girl 7 years old; had been ill for three months with a very bad cough and expectoration, emaciation and anorexia. Coarse râles were heard over both lungs. She also had a diarrhea which would not respond to treatment with the ordinary remedies. After trying many drugs for her cough, glycoheroin was given in doses of twenty drops every two hours. She also took the compound syrup of hypophosphites as a general tonic and small doses of salol and castor oil for the enteritis. Under this treatment she began to improve. Her bowels became more regular and her cough grew less severe. We did not see her for one month, and then the cough became worse. Glycoheroin was continued, twenty drops every three hours, and almost immediately some improvement showed itself. In ten days' treatment the râles had diminished and the cough was considerably better. At the time of writing the patient still had some cough, but her appetite is improving and her bowels are in good condition.

Case X.—*Asthma.*—I. W., a boy aged 8 years; has had dyspnea for several weeks, with cough and expectoration. The following mixture was given:

| | | | |
|---|---|---|---|
| ℞ | Tincture of lobelia | ʒj | 4.0 |
| | Glycoheroin | ʒv | 20.0 |
| | Syrup of hydriodic acid | ℥j | 30.0 |
| | Water enough to make | ℥ij | 60.0 |

M. Sig.—A teaspoonful every four hours.

In four days the child was very much improved. His breathing was much less labored, and he coughed very little. The medicine was continued for a week, after which the patient was no longer under observation.

Case XI.—*Bronchitis and Laryngitis.*—N. F., a girl of 6 years; had a croupy cough with hoarseness, worse at night, for five days. The following mixture was given:

| | | |
|---|---|---|
| ℞ | Syrup of ipecac | 5.0 |
| | Syrup of squills | 5.0 |
| | Glycoheroin | 8.0 |
| | Syrup of wild cherry, to make | 60.0 |

M. Sig.—A teaspoonful every three hours.

In three days the patient was considerably better, and in one week entirely well.

Case XII.—*Pertussis.*—C. V., 3½ years old; coughed for about two weeks with very violent spasms, without vomiting. Glycoheroin was given, ten drops every two hours, and later every three hours. Within three days the spasms became milder, and in two weeks more the cough had ceased entirely.

Case XIII.—*Pertussis.*—J. V., 2 years old; coughed for about two weeks, with typical paroxysms of pertussis, diarrhea, bloody stools, and temperature of 103° F. Calomel was given, followed by a bismuth mixture. Glycoheroin was given throughout the subsequent treatment in 5-drop doses. Diarrhea got well without being adversely influenced by the glycoheroin. In two weeks the cough had almost entirely disappeared.

In these cases various drugs and methods of treatment had been used without success. An older brother of these two patients had had an attack of pertussis, from which he was just recovering when we were called in to treat the other two.

Of 20 cases of cough of various kinds, 13 of which are reported in detail, 12 were cured in a short time, 7 greatly improved, and two resisted treatment and did not improve. In one of the latter, (Case VI., laryngitis and pharyngitis,) the unsuccessful result was due to irregularity in taking the medicine and to the short time of observation. In the other failure (Case V.) we had to deal with an old case of chronic bronchitis and asthma that had failed to yield to all other forms of treatment.

We must call attention particularly to the very favorable results obtained in our cases of tuberculosis and of whooping cough. In the tuberculous patients the cough had been most distressing, and was rapidly alleviated by the administration of small doses of glycoheroin. In the cases of whooping cough, the results far exceeded our expectations, which are never very high when we deal with such an intractable disease as pertussis. We found that the cough in all cases rapidly lost its spasmodic character; that the vomiting and the other distressing accompaniments of the cough ceased, and that the number and intensity of the paroxysms progressively diminished.

In two cases of asthma (Cases IV. and X.), very satisfactory results were obtained and the drug acted in an irreproachable manner in all the cases of bronchitis, except in Case VI., which has already been referred to.

CONCLUSIONS.

1. In glycoheroin we have found a perfectly satisfactory mode of administering heroin, and the results of our experience with this preparation have been such as to dictate a marked preference for it above other combinations containing the diacetic acid ester of morphine.

2. It is especially valuable in whooping cough. Small doses of it are easily borne by children, and even by infants, and the results obtained are prompt and permanent.

3. We also have found glycoheroin useful in the treatment of pulmonary tuberculosis, in reducing the cough, night-sweats, fever, pain and dyspnea; in adding to the general well being of the patient without disturbing his appetite and digestion.

4. In asthma it gives relief which compares very favorably with the effect of the depressant narcotics usually given in such cases.

5. It was always found satisfactory in acute bronchitis, while all but the most obstinate cases of chronic bronchitis yielded readily to its influence.

6. Our experience with glycoheroin in laryngitis has been too limited to warrant any conclusions as to its value in this disease.

BIBLIOGRAPHY.

1. Dreser and Floret—Therap. Monatshefte, September, 1898, p. 509.
2. Manges—New York Medical Journal, November 26, 1898.
3. Strube—Berliner klin. Wochenschr., November 7, 1898.
4. Weiss, Julius—Die Heilkunde, October, 1898.
5. Freudenthal—Philadelphia Medical Journal, March 25, 1899.
6. Lang—Medical Times and Register, March, 1899.
7. North, J.—American Medical Compend, June, 1899.
8. Goldman—Allgemeine med. Central-Zeitung, No. 33, 1899.
9. Leo—Deutsche med. Wochenschrift, March 23, 1899.
10. Beketoff—Amer. Journal of the Medical Sciences, August, 1899
11. Einhorn—Philadelphia Medical Journal, October 28, 1899.
12. Mirtl—Wiener klin. Rundschau, No. 25, 1899.
13. Herwisch—Therapeutic Gazette, November 15, 1899.
14. Fulton—New York Medical Journal, December 30, 1899.
15. Gayle—Alkaloidal Clinic, December, 1899.
16. Fluckinger—American Therapist, March, 1900.
17. Wetherby—Medical Council, November, 1899.

18. Daly—Boston Medical and Surgical Journal, February 22, 1900.
19. Medea—Charlotte Medical Journal, January, 1900.
20. Paulesco—Medical Review of Reviews, February, 1900,
21. Moody—Alabama Medical Journal, July, 1900.
22. Hotkamp—The Laryngoscope, October, 1900.
23. Brower and Tompkins—Therapeutic Gazette, August 15, 1900.
24. Wiesner—International Journal of Surgery, May, 1900.
25. Geis—New York Medical Journal, December 1, 1900.
26. Johnson—Kansas City Medical Record, May, 1901.
27. Pawinski and Adelt—Heilkunde, January, 1901.
28. Sickler—Medical Age, January 25, 1902.
29. Conner—Medical Standard, January, 1902.
30. Loewenthal, M.—Canadian Journal of Medicine and Surgery, May, 1901.
31. Johnson—Amer. Pract. and News, December 1, 1900.
32. Hyams—Medical News, December 1, 1900.
33. Lazarus—Boston Medical and Surgical Journal, December 13, 1900.
34. Loewenthal—Gaillard's Medical Journal, April, 1901.
35. Gillies—Montreal Medical Journal, June, 1901.
36. Mitchell—Virginial Medical Semi-monthly, November 16, 1900.
37. Freudenthal—Journal of the American Medical Association, March 16, 1901.
38. Martinson—Medical Times, January, 1901.
39. Helbich—Die Heilkunde, May, 1902.
40. Ashby—Medical Review of Reviews, November, 1901.
41. Love—Medical Mirror, January, 1902.
42. Levien—Buffalo Medical Journal, September, 1901.
43. De Lorme—Interstate Medical Journal, 1901.

